# Targeting the cMET pathway to enhance immunotherapeutic approaches for mUM patients

**DOI:** 10.3389/fonc.2022.1068029

**Published:** 2023-01-24

**Authors:** Devayani Machiraju, Jessica C. Hassel

**Affiliations:** Department of Dermatology and National Center for Tumor Diseases (NCT), University Hospital Heidelberg, Heidelberg, Germany

**Keywords:** uveal melanoma, immunotherapy, liver metastasis, cMET signaling, combination (combined) therapy, PD1 (programmed cell death protein 1), LAG3: Lymphocyte-activation gene 3, resistance mechanism

## Abstract

The liver is the most preferential initial site of metastasis for uveal melanoma (mUM), and this preference is associated with rapid mortality in mUM patients. Despite the significant clinical benefits of Immune checkpoint inhibitors (ICIs) in metastatic cutaneous melanoma patients, ICIs have shown little to no benefit in mUM patients. A potential reason for this inefficiency of ICI could be partly devoted to the involvement of the liver itself, thanks to its rich source of growth factors and immunosuppressive microenvironment. Uveal melanoma cells show increased expression of a transmembrane protein called cMET, which is known as the sole receptor for the Hepatocyte growth factor (HGF). Hyperactivation of cMET by HGF contributes to mUM development, and the liver, being the major source of HGF, may partially explain the metastasis of uveal melanoma cells to the liver. In addition, cMET/HGF signaling has also been shown to mediate resistance to ICI treatment, directly and indirectly, involving tumor and immune cell populations. Therefore, targeting the cMET/HGF interaction may enhance the efficacy of immunotherapeutic regimes for mUM patients. Hence in this minireview, we will discuss the rationale for combining cMET inhibitors/antibodies with leading immune checkpoint inhibitors for treating mUM. We will also briefly highlight the challenges and opportunities in targeting cMET in mUM.

## 1 Introduction

Melanocytes have functional roles in light absorption, regulation of oxidative stress, and the immune system (1). Compared to skin melanocytes, melanocytes in the eye evolved with distinct biologic characteristics, gene expression profiles, and immune regulatory pathways (2–6). Uveal melanoma (UM) is a rare but deadliest cancer that develops from the melanocytes in the uveal tract of the eye (7). The most intriguing features of UM include 90% of tumors harboring a mutation in GNA11 and GNAQ genes and its extraordinary preference to spread and colonize in the liver. As a result, approximately 90-95% of metastatic UM tumors will develop liver metastasis (LmUM), unlike metastatic cutaneous melanoma, which is only observed in approximately 20% of patients clinically (8–10). This preference is not only associated with rapid mortality with a survival expectancy of less than a year but also confers resistance to many anti-cancer therapies, including immunotherapy (11). Immunotherapy with immune checkpoint inhibitors (ICIs) such as anti-cytotoxic T-lymphocyte associated protein 4 (anti-CTLA4) and anti-programmed cell death protein 1 (anti-PD1) reverse the exhausted anti-tumor immune cell responses and has given a tremendous hope in terms of survival for metastatic cutaneous melanoma (mCM) patients in the clinic (12, 13). However, the same cannot be stated for metastatic uveal melanoma (mUM) patients (14). This inefficiency can be partly due to the involvement of the liver itself, as the ICI treatment has also been shown to be less efficient in mCM patients with liver metastasis (15, 16).

The preferential spreading of UM to the liver cannot be explained just by the anatomical connection but by biological attraction and immunosuppressive microenvironment of the liver that can protect the UM growth in this distant organ once spread. Molecular communication seems pivotal as UMs overexpress specific protein receptors for which the growth factors derived from the liver may serve as ligands, hence acting as chemoattractants that direct UM to the liver (17). Moreover, as the liver interacts with various foreign antigens, it tightly regulates immune responses to avoid unwanted immune reactions, which UM can exploit to promote metastasis (18). *In vivo*, the CD27+CD11b- immature natural killer cells in the liver were shown to promote the development of melanoma metastasis to the liver (19). Reduced infiltration of PD1+ and CTLA4+ T cells was observed in mice with liver metastasized melanoma compared to mice with only subcutaneous melanoma, suggesting that liver metastasis may also regulate systemic immune responses (20). Besides, direct evidence of tumors from melanoma patients with liver metastases has revealed reduced infiltration of CD8+ T cells and PD1+ T cells but increased infiltrations of TIM3+ T cells. Together these data suggest that tumors metastasized to the liver may experience a unique microenvironment (21). Throughout this process, strategies targeting immune suppression with ICIs alone seem insufficient for handling mUM; hence we may have to explore and evolve treatment combinations that can potentially target tumor signaling pathways involved in LmUM development along with immune-enhancing regimes. In this regard, the hepatocyte growth factor receptor (cMET) signaling pathway has been extensively studied and reviewed for its role in developing LmUM and may potentially enhance ICI treatment.

cMET is a transmembrane protein composed of α and β subunits connected by disulfide bonds at the N-terminus. The α subunit remains extracellular, whereas the membrane-spanning β subunit consists of extracellular, transmembrane, and an intracellular tyrosine kinase domain at the C-terminus (22). It is usually expressed in a wide range of cells, including immune cells such as neutrophils, and is involved in cellular processes like survival and migration. So far, there are four ligands reported for the cMET receptor, hepatokines such as hepatocyte growth factor (HGF), leukocyte cell-derived chemotaxin-2 (LECT2), decorin, a small leucine-rich proteoglycan, and a bacterial surface protein called Internalin B (IntlB) from Listeria Monocytogenes which is needed for pathogen entry into the host cell (23–26). All four proteins are known to bind and interact with the extracellular region of cMET but with different binding affinities. The binding of HGF or IntlB results in cMET phosphorylation and activation of signaling pathways that regulate cell proliferation, epithelial-to-mesenchymal transition (EMT), and anti-apoptotic effects. In contrast, LECT2 and decorins can counter the HGF and Intl B signaling (23, 26). For example, LECT 2 binding inhibits cMET phosphorylation and Raf1/ERK signaling, which is responsible for EMT transition, and its absence promotes EMT in tumoral hepatocytes (27, 28). Similarly, the binding of cMET to decorins expressed in most extracellular matrices suppresses intracellular beta-catenin levels and inhibits cMET-mediated cell migration and growth (29). cMET/decorin binding also promotes rapid intracellular degradation of cMET *via* the recruitment of the E3 ubiquitin ligase c-Cbl (25).

Meanwhile, considering its significant role in cell proliferation and migration, it is not surprising to see cMET upregulation in cancer cells, but interestingly the threshold of its overexpression is way higher in mUM compared to other tumors, including mCM (30). Loss of cMET negative regulators, gene amplification, or germline mutations in exon 14 of cMET may result in such altered gene expression in tumors (31–33). The high-affinity binding of liver-derived HGF ligand to cMET can drive LmUM (23). *In vitro*, cMET/HGF signaling induces FAK/MAPK/STAT signaling pathways and regulates tumor cell growth invasion. cMET/HGF signaling also induces AKT/mTOR signaling pathways that activate E3 ubiquitin ligase MDM2, inhibiting apoptosis. Whereas cMET inhibition or downregulation of cMET suppresses UM proliferation and migration by inhibiting these pathways *in vitro* and *in vivo* (34). Consistent with its role in migration, liver metastatic lesions from UM patients have shown increased expression of cMET compared to primary tumors (35). In addition, over-expression of cMET correlates with high-risk parameters in UM and indicates a poor prognosis (36). cMET signaling is also involved in tumor resistance to many cancer treatments (37–40). Therefore, targeting cMET is a promising area of cancer drug development, and cMET targeting approaches are currently being investigated in clinical trials for various cancers, including mUM.

## 2 cMET inhibitors in clinical trials for advanced cancer patients, including mUM

**Figure 1:**
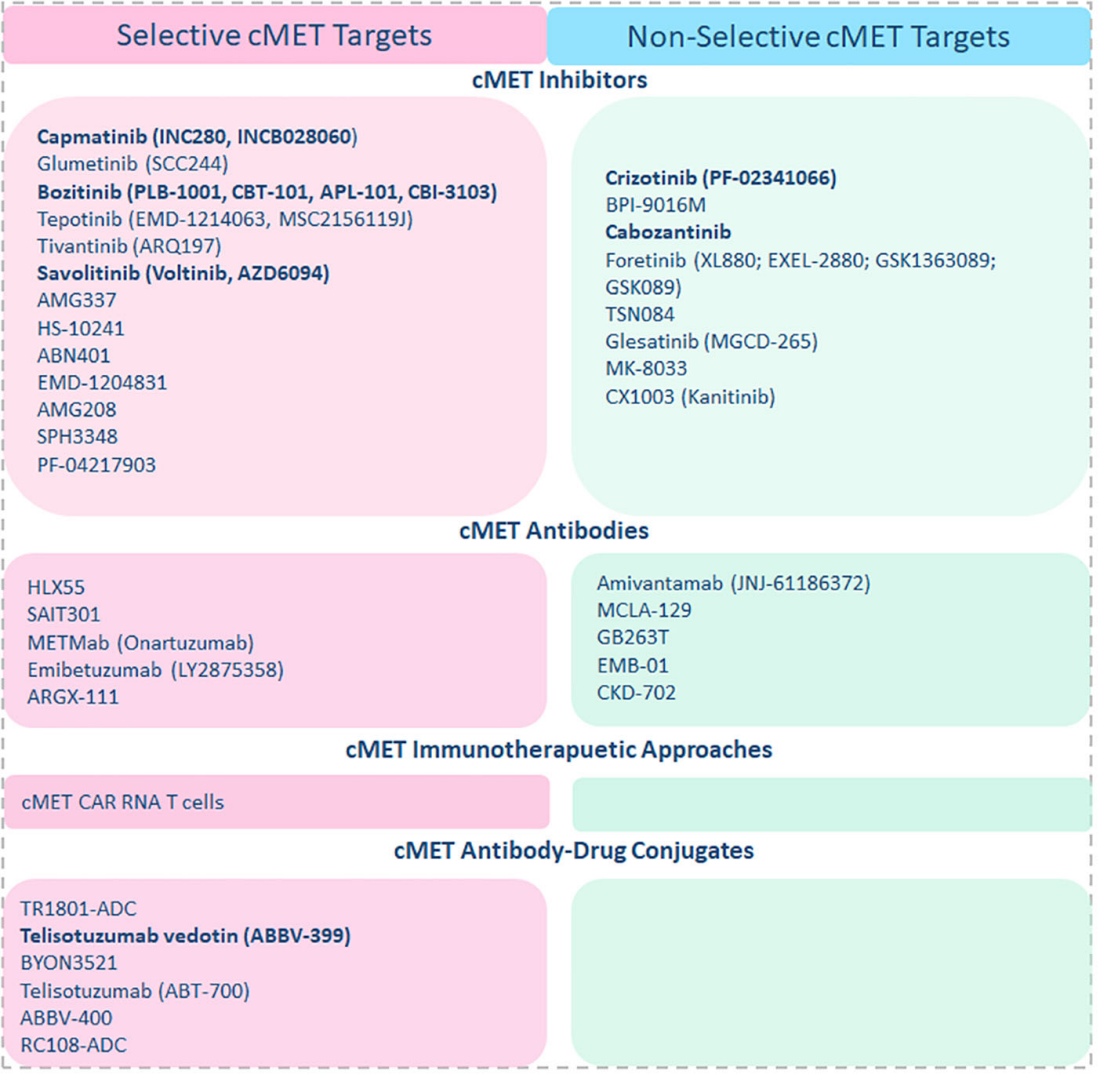
cMET-targeting approaches and agents in clinical trials are listed accordingly, and cMET-targeting agents currently being investigated in combination with ICI regimes are highlighted in the figure.

So far, many strategies to target cMET, including immunotherapeutic approaches such as cMET-specific CAR T cells, cMET neutralizing antibodies, and small molecule inhibitors, are already being tested in clinical trials of solid tumors (**Figure 1**). Some have already demonstrated impressive therapeutic efficacy in the clinical setting. Intra- tumoral injection of cMET targeting CAR T cells was well tolerated in breast cancer patients, resulting in tumor necrosis and loss of cMET immunoreactivity (41). At the same time, a wide range of cMET small molecule inhibitors are currently being investigated in clinical trials for several cancers, including mUM. cMET inhibitors can be classified as selective inhibitors that only target cMET and non-selective inhibitors that target cMET along with other pathways such as VEGF, ALK, etc. Both have shown significant anti-tumor activity in a wide range of cancers and manageable safety profiles in the clinic. Tepotinib, a highly selective oral cMET inhibitor, was well tolerated, has shown promising efficacy in advanced hepatocellular carcinoma patients with cMET overexpression, and is currently being investigated for other cMET-deregulated cancers (42, 43). Tivantinib, another selective cMET inhibitor in combination with Sorafenib, a multi-kinase inhibitor, has also shown promising results in clinical trials of cancer patients, including advanced melanoma with a 63% of disease control rate. Interestingly, in this study, cMET overexpression was noted in only 29% of patients, and all patients with increased cMET expression have been shown to respond to the treatment (44). Meanwhile, two clinical trials were registered or are currently ongoing with cMET inhibitors for mUM. Among them, a phase II clinical trial including heavily pre-treated mUM patients who received a combination of Crizotinib, a non-selective cMET inhibitor, and Darovasertib has shown to achieve a 100% disease control rate and 31% response rate according to the preliminary reports from the investigators (45). Treatment-related adverse events (AEs), including 54% of grade 1 or 2 and 27% of patients with grade 3, were reported. In line with these findings, a reanalysis of a discontinued phase 2 trial revealed that cabozantinib, a multi-kinase inhibitor that targets cMET along with AXL, and VEGFR2, displayed anti-tumor activity with 61% disease control rate and a median PFS of 4.8 months in mUM patients (46). Although the current clinical trials targeting cMET are conducted mostly in combination with other kinase inhibitors making it hard to interpret the sole efficacy of cMET inhibition, collectively, these data suggest that cMET inhibitors are safe and can show anti-tumor activity in mUM patients.

Nevertheless, similar to the fate of other small molecule inhibitors, the mUM may acquire resistance under cMET inhibitors. Besides, the constant TCR signaling or the highly immunosuppressive tumor microenvironment may soon lead to the exhaustion of cMET inhibition-induced anti-tumor immune responses (47). Therefore, even though the exact mechanisms of their additive activity are yet to be proven in mUM, there is a strong rationale for cMET inhibitors to evolve with ICI regimes. To this point, most of the successful attempts of ICI usage in cancers like liver cancer were demonstrated in combination or when pre-treated with small molecule tyrosine kinase inhibitors, suggesting the potential of cMET inhibitors when evolved in combination with ICIs in mUM patients (48, 49).

## 3 cMET inhibitors in combination with Immunotherapy

**Figure 2:**
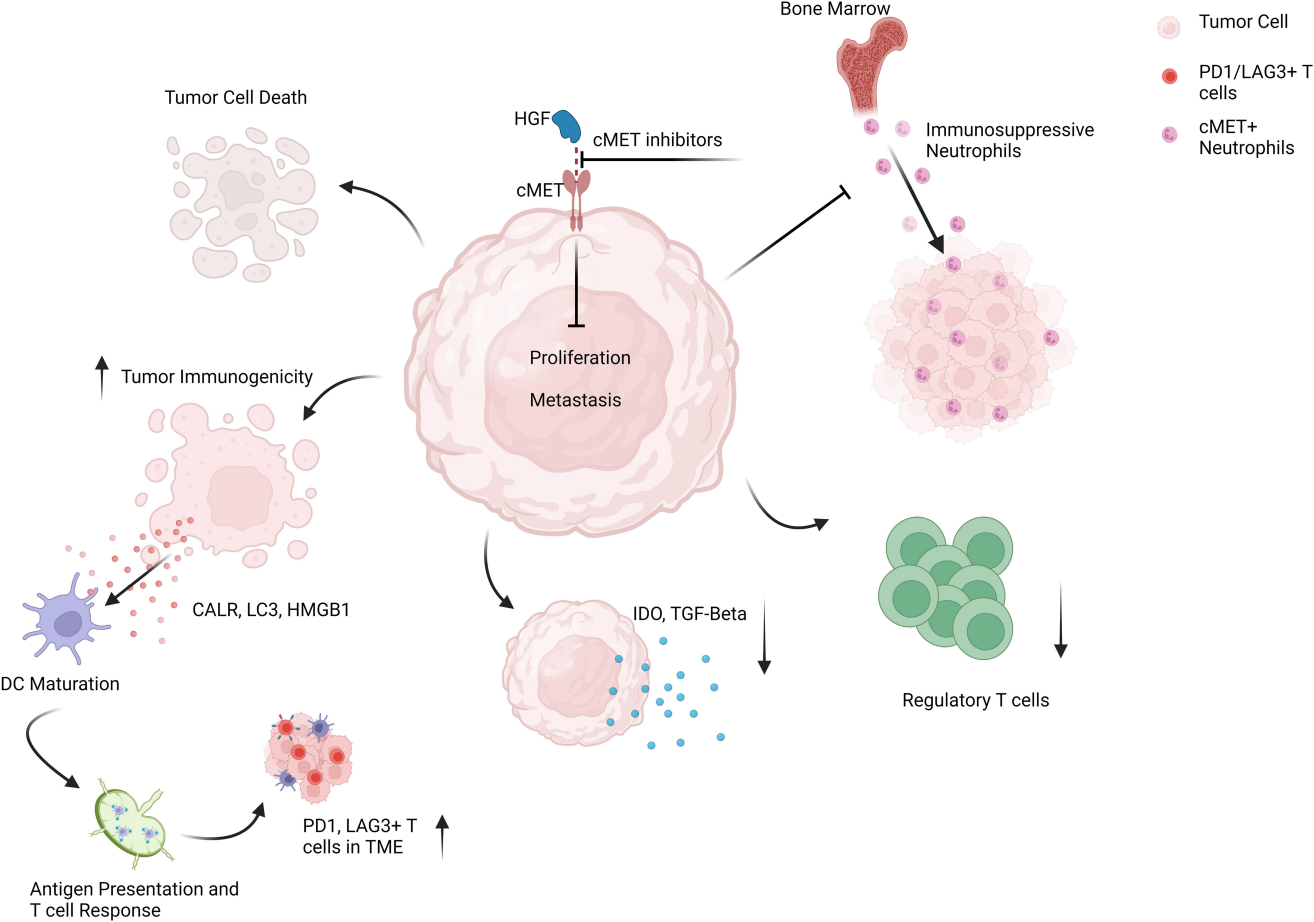
Schematic representation of targeting cMET pathway in mUM patients to enhance the anti-tumor efficacy of ICI regimes.

Anti-PD1 monotherapy has shown minimal success in mUM patients, with responses ranging from 0-7% with a median PFS of 2-3 months and OS limited to approximately one year (50, 51). Increased hepatic tumor load and intrinsic tumor factors such as low tumor mutational burden, PD1/PDL1 expression levels, and increased presence of alternative immunosuppressive molecules like Lymphocyte-activation gene-3 (LAG3) in mUM compared to mCM may explain such inefficacy (52–55). Interestingly, the cMET pathway is also involved in resistance to anti-PD1 treatment and associated with almost all factors mentioned above, strengthening the rationale for combination treatment (**Figure 2**). cMET expression correlates with liver metastasis of UM and tumor mutational burden in lung cancer patients, and cMET/HGF signaling has been shown to upregulate indoleamine-2,3-dioxygenase (IDO) 1, a highly immunosuppressive molecule involved in resistance to immunotherapy (35, 56–58). cMET inhibition stops tumor growth and invasion and can improve anti-PD1 response by involving immune cells. High-dose crizotinib induces immunogenicity in tumor cells and increases the expression of PD1, LAG3, and PDL1, in a mouse model for lung cancer, therefore preparing the tumors for immunotherapy with anti-PD1 antibodies (59). In addition, neutrophils can play a significant role in the promotion of tumor growth and liver metastasis, and their increased presence in the blood or tumor is another factor known to contribute to anti-PD1 resistance (60–63). *In vivo*, HGF/cMET signaling drives neutrophils from bone marrow to the TME, where they acquire immunosuppressive properties in T cell inflamed tissues and suppress immunotherapy-induced CD8+ T cell expansion and effector function. Meanwhile, concomitant inhibition of cMET and PD1 blocked the infiltration of bone marrow-derived immunosuppressive neutrophils into the tumor and led the way for the anti-tumor efficacy of PD1 inhibitors in mouse tumor models (64). Accordingly, a novel dual inhibitor of cMET and PD1 has shown superior anti-tumor efficacy with solid anti-proliferative and anti-metastatic effects compared to monotherapy with PD1 inhibitors alone in tumor cell lines and in a mouse model with liver cancer (65). Besides, several clinical trials are currently evaluating the safety and efficacy of cMET inhibitors in combination with ICIs for advanced cancer patients. A clinical phase I/II combination therapy with Cabozantinib and Nivolumab in advanced liver cancer patients reported a disease control rate of 81%, a response rate of 17%, and a median PFS of 5.5 months. However, a triple combination therapy including Cabozantinib, Ipilimumab, and Nivolumab has shown higher response rates but had to be discontinued due to increased treatment-related toxicities (66). Likewise, this combination showed a better response rate, delayed disease progression, and extended patient survival in treatment-naive metastatic renal cell carcinoma, another cMET-deregulated tumor (48). Collectively, these data suggest that a concomitant combination of cMET inhibitors and ICIs, or a sequential combination of cMET inhibitors followed by ICIs, may improve responses to cancer immunotherapy on multiple fronts in mUM patients.

Like PD1, LAG3 is also an inhibitory receptor that controls T-cell tolerance and is the next most promising immune target after PD1 (67). It is particularly promising for mUM because it functions as an alternative pathway for PD1, and LAG3-positive T cells are more abundant in mUM TME than CTLA4 or PD1-positive T cells (54, 55). Furthermore, LAG3 is highly expressed in monosomy of chromosome 3 (M3) UM tumors and, similar to cMET, correlates with high-risk histopathological parameters (68). Accordingly, increased expression of LAG3 in TILs linked to reduced disease-free survival in mUM patients (68). Relatlimab, an anti-LAG3 antibody, has already shown promising clinical benefits in mCM patients in combination with the PD1 antibody nivolumab (69), and clinical trials are currently ongoing to determine the efficacy of relatlimab plus nivolumab in mUM patients (NCT04552223); therefore, a combination of cMET and LAG3 inhibitors theoretically offers another potential strategy for mUM. However, further studies are required to understand the relationship between these two proteins in mUM tumors and the consequences of blocking them together. Similarly, tebentafusp is the most promising soluble TCR molecule offering survival benefits in mUM patients with HLA-positive tumors (70). Even though patients progressing under tebentafusp have a survival benefit, patients responding to treatment have better overall survival. However, response rates are low, and strategies to increase efficacy through combination therapies are of great interest. cMET inhibitors can increase the immunogenicity of tumors and may further lead to an additive benefit for tebentafusp (59).

## 4 Challenges & opportunities

Although there are abundant possibilities for combining cMET inhibitors with ICIs, several important tasks lie ahead to move the combination treatment from the lab to the clinic. Firstly, there is a need to explore the most selective and active cMET inhibitor with less off-target toxicities and high anti-tumor activity and then to evaluate the best ICI agent (anti-PD1 or/and anti-LAG3) to combine for mUM treatment. Although anti-PD1 antibodies are standard care, given the strong rationale for targeting LAG3 in mUM, evaluating the combination efficacy of cMET inhibitors and anti-LAG3 antibodies in mUM patients is highly demanding. Bispecific antibodies targeting anti-cMET/PD1 or anti-cMET/LAG3 are also promising developments for mUM. Secondly, further preclinical research aiming to design the best sequence of combination treatments, cMET inhibition followed by ICI or a concomitant inhibition, should be investigated thoroughly to integrate the combination treatments effectively in well-designed clinical trials. Consequently, a crucial task is also to identify the patients who may benefit from such combination treatment strategies. Identifying cMET overexpressed tumors may serve this purpose. However, the scarcity of tissue material and the tumor heterogeneity might make it challenging to identify the cMET-positive tumors and to define a cut-off for its overexpression. Systemic biomarkers can overcome these limitations and serve as a non-invasive material to investigate predictive biomarkers. In this regard, higher concentrations of soluble cMET, a cleaved product of membrane cMET, have been detected in the blood and may indicate the overexpression of cMET in tumors from mUM patients. Accordingly, increased concentrations of soluble cMET have been shown to correlate with worse survival in mUM patients suggesting its potential in identifying cMET tumors (71). Likewise, the abundance of circulating cMET-positive tumor cells captured using novel cMET-based Ferrofluid also be an alternative method (72). Finally, cMET-based PET CT has also been developed recently and is currently being tested in renal cell carcinoma patients; adopting such screening procedures for mUM patients can identify the mUM patients with cMET-positive tumors (73).

## 5 Conclusions

Enhancing the efficacy of ICI treatment for mUM patients is highly demanding. The major challenge here is the involvement of the liver in mUM patients. Targeting signaling pathways involved in liver metastasis may improve the anti-tumor efficacy of ICIs. Inhibition of cMET, a transmembrane protein highly involved in LmUM progression, induces tumor cell death and inhibits signaling pathways in tumor resistance. In addition, cMET inhibition also increases tumors’ immunogenicity and blocks immune-regulating neutrophils’ infiltration into TME, therefore, sensitizing the tumors to ICIs. Accordingly, ICIs combined with cMET inhibitors are currently being investigated in clinical trials for other cancer types and have already shown promising results in patients with liver cancer. Therefore, applying the knowledge from these studies and developing combined treatment strategies, including cMET inhibitors and ICIs for mUM patients, may have tremendous clinical potential. While the benefit of such combination therapy may sound substantial in terms of clinical benefits, several issues must be addressed to adapt this approach safely and efficiently for mUM patients. Selecting a potential cMET targeting agent or approach, identifying predictive biomarkers, and the best treatment sequence for combination should be thoroughly investigated. Besides, as combination therapies are highly associated with increased toxicity compared to monotherapies, the mechanism underlying the synergistic effect must be clarified in preclinical models. Hence, further research efforts are urgently required to optimize the development and delivery of cMET-ICI combinations, and it is highly anticipated that future studies on this combinational approach will lead to survival benefits for mUM patients.

## Author contributions

DM and JH conceived and designed the overall study. DM prepared the original manuscript. JH supervised, reviewed and approved the final manuscript. All authors contributed to the article and approved the submitted version.
